# Readiness of physicians and medical students to cope with the COVID-19 pandemic in the UAE

**DOI:** 10.1371/journal.pone.0251270

**Published:** 2021-05-06

**Authors:** Hiba J. Barqawi, Drishti D. Kampani, Enad S. Haddad, Nora M. Al-Roub, Eman Abu-Gharbieh

**Affiliations:** 1 Department of Clinical Sciences, College of Medicine, University of Sharjah, Sharjah, United Arab Emirates; 2 College of Medicine, University of Sharjah, Sharjah, United Arab Emirates; Jouf University, Kingdom of Saudi Arabia, SAUDI ARABIA

## Abstract

**Background:**

Coronavirus disease (COVID-19), caused by Severe Acute Respiratory Syndrome–Coronavirus 2 (SARS-CoV2), is the defining global health crisis of this time. It is responsible for significant morbidity and has had severe socioeconomic consequences. This study aims to assess the knowledge, preparedness and attitudes of medical students, physicians and faculty members in the United Arab Emirates (UAE) on COVID-19 and their perspective on the roles of educational and healthcare institution towards improving pandemic preparedness and enabling optimal care.

**Methodology:**

An exploratory, descriptive cross-sectional study was conducted with 444 participants, using a non-probability convenience sampling method. English-speaking participants from the medical field aged 18 and above were included in the study. The validated questionnaire was administered online and distributed across social media platforms from May-July 2020. T-test, ANOVA, Kruskal-Wallis test and Mann-Whitney-U test were used when appropriate. Responses were analysed and statistical tests applied using IBM SPSS, version 25.

**Results:**

The knowledge scores were calculated amongst different ages and professional status, and the mean was 59.08% (SD = 12.848%). Almost half of the participants obtained poor knowledge scores (less than 60%). Most of the participants followed the latest updates on COVID-19 (86.7%). The majority opted to obtain information from the national health authorities (63.4%). The mean preparedness score among the participants was 68.65% (SD = 17.456%). Being in contact with patients significantly increased the preparedness score (*p* < 0.001). Only 27.9% of the participants believed their college education provided adequate knowledge to deal with epidemics or pandemics. Several barriers affect willingness to work in a pandemic, with 80.6% of participants worried about posing a risk to family members.

**Conclusion:**

This study highlights the importance of establishing tailored COVID-19 related education programs to improve knowledge levels, especially in medical students. Efforts are still needed to promote effective control measures and address the barriers affecting willingness to work in a pandemic.

## Introduction

The Coronavirus Disease-19 (COVID-19) pandemic has firmly established itself as the defining global health crisis of this time and one of the greatest challenges that we continue to face as a global community. As of 12^th^ March 2021, over 118,268,575 cumulative cases and 2,624,677 deaths have been reported globally since the beginning of the outbreak, with a case fatality rate of 2.22% [1]. In the United Arab Emirates (UAE) alone, 422,246 cumulative cases were identified as of 12^th^ March 2021, with a case-fatality rate of 0.30%, making it an urgent national health concern [1].

Severe acute respiratory syndrome coronavirus-2 (SARS-CoV-2) has been identified as the causative agent for COVID-19 [2]. The main routes of transmission of this virus are through respiratory droplets or contact with contaminated surfaces [2]. The disease has an average incubation period of 5–6 days, extending up to 14 days, with variable manifestations ranging from asymptomatic to serious, life-threatening symptoms among certain individuals. The most common symptoms experienced are fever, cough, and fatigue. In severe cases, the disease develops to progressive respiratory distress syndrome and multi-organ failure [3, 4].

As of March 2021, several immunosuppressive and anti-viral therapies have been approved to manage cases of COVID-19. However, there was no consensus on approved treatment in the early months of 2020, and therefore, prevention was the main approach to tackle this health crisis. Preventive measures provided by the World Health Organization (WHO) include the usage of face masks, regular handwashing with soap or disinfection with alcohol-based sanitisers, and maintaining a distance of at least 1 meter (3 feet) from other people [5].

Healthcare workers, primarily physicians, have been at the forefront of responding to the pandemic, constantly adapting to changing information and coordinating efforts to build the strength of a country’s healthcare system. They play a vital role through screening, diagnosing, monitoring, triaging, and accommodating the patients with the most recent treatment plans. Furthermore, they provide safety precautions to the public that must be based on reliable scientific facts [6].

Several studies conducted during previous pandemics highlighted the knowledge gap among healthcare professionals, which in turn would impact willingness to work [7–9]. However, there is not enough published research shedding light on the knowledge, attitudes and barriers to working for essential healthcare workers in the COVID-19 crisis, especially in the UAE. It is important to recognise that healthcare workers are the primary sector in contact with COVID-19 patients; they are ultimately expected to be at higher risk to acquire the infection in comparison to the normal population. The WHO and the Centers for Disease Control and Prevention (CDC) have provided and published guidelines to follow in the healthcare setting [10, 11]. Physicians and medical students are already participating in providing care to COVID-19 patients [12]. Moreover, faculty members play a role in conveying accurate information to medical students. Thus, accurate knowledge and suitable practices are integral in managing and eradicating the virus in addition to dealing with future pandemics.

This study aims to explore the knowledge, preparedness and attitudes of medical students, physicians and faculty members in the UAE during the current COVID-19 pandemic, as well as their perspective on the roles of educational and healthcare institutions in improving pandemic preparedness.

## Methodology

### Study design

An exploratory, descriptive cross-sectional design was used in this study. The questionnaire was developed on Google Forms and distributed to medical professionals above the age of 18 years; this included faculty members and students from all Colleges of Medicine in the UAE, as well as medical interns, resident physicians, general practitioners, specialists, and consultants licensed by the health authorities in the country. In addition, participants from all seven emirates were recruited. The questionnaire was designed in English; thus, non-English speakers were not compatible candidates. Furthermore, participants in non-medical fields and healthcare fields other than medicine were excluded. The study was approved by the Research Ethics Committee, University of Sharjah, with *reference* No REC-20-06-03-01.

### Sample and data collection

Sample size calculation with a confidence level of 95%, margin of error of 5%, and study power of 80% determined the minimum required sample size to be 373 participants. A primary questionnaire was piloted to ensure conciseness and clarity. It was also sent to a group of experts to ensure content, reliability, and face validity. Once feedback was received, a final modification of the content was carried out, followed by a second pilot study. The questionnaire link was sent to the targeted population through email and social media (Instagram and WhatsApp). The distribution of the questionnaire was executed from May through July 2020. Participant information sheet (PIS) was presented before starting the questionnaire, and agreement to fill the questionnaire indicated the consent of the participants to join the study. The first question in the questionnaire offered recipients electronic, written consent to participation. Additionally, the collected data was available only to the investigators to ensure confidentiality. Distribution of the questionnaire to individuals within the inclusion criteria was carried out through several social media outlets, and a non-probability convenience method was utilised through an online format. For instance, a larger audience was reached by sharing the online questionnaire through emails sent to acquaintances within the medical field or known clinical board members. Class representatives of different age groups and universities around the UAE were sent links through Instagram and WhatsApp direct messages or groups. Clinical tutors and physicians were also contacted through WhatsApp, email, and Instagram.

### Questionnaire development

This questionnaire was designed to assess the knowledge, preparedness, and attitude of people within the medical field on the COVID-19 pandemic. In the absence of validated tools, the questionnaire was developed from the latest updates on the disease and similar studies conducted in other parts of the world. The questionnaire consists of 67 questions and is divided into eight main sections: (1) Socio-demographics, (2) Perception of knowledge of previous pandemics, (3) Knowledge of COVID-19 as a disease, (4) Preparedness to deal with COVID-19, (5) Attitudes and Behaviours during COVID-19, (6) Sources of COVID-19 knowledge, (7) Perception regarding roles of colleges of medicine and health authorities, and (8) Barriers and incentives affecting willingness to work in a pandemic. These sections used a combination of different question designs such as 5-item Likert scales, multiple-choice, and true or false questions.

### Variable management

Three of the eight sections–knowledge, preparedness, and attitudes–involved the development of respective scoring systems, elaborated as follows.

#### Knowledge score

A total of 16 questions assessed knowledge on COVID-19. There were 2 multiple-choice questions–signs and symptoms (with 11 items, of which 7 were marked as correct), modes of transmission of COVID-19 (with 7 items, of which 4 were marked as correct). Each correct item selected by the participant was awarded 1 point. The remaining 14 questions were of true-or-false type and correct identification was awarded 1 point. No negative scoring was used to penalize wrong answers.

Hence, a knowledge score of maximum 25 points was designed. The score was then converted to percentages and classified as Good (80–100%, 20–25 points), Moderate (60–80%, 15–19 points), or Poor (<60%, 0–14 points) based on Bloom’s cut off points [13].

#### Preparedness score

A series of 5 questions were designed to assess their level of confidence in dealing directly with COVID-19 patients–as frontline workers. A preparedness score was then generated by assigning value to the Likert scale ("Not at all confident" = 1 point, "A little confident" = 2 points, "Somewhat confident" = 3 points, "Fairly confident" = 4 points, "Highly confident" = 5 points). A total of 25 points was allocated to the five questions and a preparedness score out of 25 was designed. The score was then classified as Good (80–100%, 20–25 points), Moderate (60–80%, 15–19 points), or Poor (< 60%, 0–14 points) based on Bloom’s cut off points [13].

#### Attitude score

A series of six ’Yes or No’ questions were asked to assess the behaviour of the participants during the pandemic. One point was given to each favourable attitude, and an attitude score out of 6 total points was generated. Bloom’s cut off points was used to classify attitude status into favourable attitudes (80–100%; 4.8–6.0 points), neutral attitudes (60–80%, 3.6–4.7 points), and unfavourable attitudes (<60%, 0–4.6 points).

### Data analysis

Data was imported from Microsoft Excel to IBM SPSS, version 25 (IBM Corp., Armonk, NY, USA), for analysis and interpretation. Socio-demographics comprised questions about sex, age, nationality, professional status. Frequency distributions were calculated for categorical variables; means could not be calculated from a Likert scale; thus, the mode was calculated for these questions. Other scale data, such as knowledge and preparedness scores, were assessed by calculating the mean and median. When appropriate parametric assumptions were met, the T-test and ANOVA tests were utilised (followed by post-hoc tests); otherwise, non-parametric tests were used–specifically the Independent Samples Kruskal-Wallis test and Independent Samples Mann-Whitney-U test. The variability in knowledge score and preparedness score was observed through stepwise multiple linear regression analysis. Categorical variables with more than two categories were transformed into dummy variables. Preceding data analysis, assumptions of all multiple linear regression was performed. Consequently, collinearity was tested using bivariate correlation analysis as well as tolerance and variance inflation factor. Cook’s distance was used to identify outliers, whereas the Durbin-Watson test was conducted to assess the independence of observations, and finally, a P-P plot was used to quickly visualise the relations.

## Results

### Sociodemographic data of the study population

The sample size required for the study was achieved. In total, 507 responded to the questionnaire and 448 completed the questionnaire (completion rate = 88.4%). However, a final number of 444 participants remained due to excluding two participants who were dentists and two who were pre-medical students. The demographics of the study sample are demonstrated below in Table 1‎. Out of the 444 participants, 298 were females, and 146 were males, with a mean age of 24.2 (SD = 8.96; range = 18–67) years and a median age of 22.0 years. Most participants fell in the 18–24 years age group (82.2%).

**Table 1:** Demographic data of study population.

		Number (n)	Valid Percent (%)
**Sex**			
	Female	298	67.1
	Male	146	32.9
**Age Groups**	18–24 years	365	82.2
**Mean: 24.24 (SD = 8.956 years)**	25–35 years	36	8.1
	36–45 years	16	3.6
	>45 years	27	6.1
**City of Residence**			
	Abu Dhabi	84	18.9
	Dubai	134	30.2
	Sharjah	192	43.2
	Northern Emirates	34	7.7
	Ajman	13	2.9
	Umm Al Quwain	13	2.9
	Ras Al Khaimah	1	0.2
	Fujairah	7	1.6
**Nationality**			
	UAE National	89	20.0
	Arab Expatriate	278	62.6
	Non-Arab Expatriate	77	17.3
**Professional Status**			
	Pre-clinical Phase Student (Years 1–3)	224	50.5
	Clinical Phase Student (Years 4–5)	80	18.0
	Recent Graduates/ Interns	65	14.6
	Resident Physician	15	3.4
	Physician/ Specialist/ Consultant	31	7.0
	Faculty Member	29	6.5
**Years of clinical experience**			
	0 years	257	57.9
	1–5 years	146	32.9
	6–10 years	7	1.6
	> 10 years	34	7.7
**Patient Contact**			
	Yes	131	29.5
	Everyday	56	12.6
	4–6 days per week	44	9.9
	1–3 days per week	23	5.2
	Not specified	8	1.8
	No	313	70.5
**Clinical workshops attended in the past year**			
	None	116	26.1
	One	85	19.1
	Two	105	23.6
	Three	47	10.6
	Four	33	7.4
	Five	11	2.5
	> 5	47	10.6

### Perception of knowledge of previous epidemics and pandemics

Of all the participants, 73.9% stated that they had received enough previous education regarding previous epidemics and pandemics, but only 52.0% were able to correctly distinguish the definition of an epidemic from that of a pandemic or an outbreak.

The participants were also asked about their primary source of knowledge on previous epidemics and pandemics. Colleges of Medicine (46.2%) was the most frequently used source of information, followed by published articles and research (13.3%). Previous knowledge on epidemics and pandemics showed a significant inverse association with COVID-19 knowledge score (*p<*0.045*);* those with poor previous knowledge had higher COVID-19 knowledge scores.

Additionally, they were asked to rate their level of agreement with the statement that ’College education adequately provided them with the knowledge to deal with epidemics and pandemics.’ It was found that 31.1% of the participants disagreed, 41.0% were neutral, and only 27.9% agreed with that statement.

### Knowledge of COVID-19 as a disease

The knowledge score of all study participants ranged from 0 to 100%, with a mean of 59.08% (SD = 12.848) and a median of 60.00% (IQR = 16). Most participants (46.8%) obtained poor knowledge scores, whereas only 7.9% had a good knowledge score.

A vast majority of the participants were able to identify the most commonly experienced symptoms in COVID-19 patients correctly; fever (98.2%), cough (96.4%), and shortness of breath (94.2%). Moreover, there seemed to be a noticeable confusion identifying other less common symptoms such as myalgia (38.7%), sputum production (15.1%), and anorexia (14.4%). However, most participants were able to correctly differentiate incorrect or false symptoms.

As for the modes of transmission, 96.8% correctly identified the exposure of the nose or mouth to infected respiratory droplets as a main method of transmission (S1 Table).

Nearly half of the participants (55.2%) knew that a positive antibody test does not necessarily indicate a current infection. However, only 32.0% knew that a negative COVID-19 RT-PCR does not always translate to a patient being free of the pathogen. The majority (46.6%) did not know that lymphopenia is associated with COVID-19.

Most of the participants (70.9%) correctly recognised that acute respiratory distress syndrome and acute tubular necrosis are common complications of COVID-19. 71.6% understood that hand washing with soap and water for 20 seconds is a good practice to protect against the spread of infection. But, 40.3% agreed that respirator (N95 mask) and gloves are sufficient to deal with suspected or confirmed COVID-19 patients, when that statement is not true.

Majority (88.1%) knew that there is no current approved treatment for COVID-19 management. Nearly 43.5% did not know or incorrectly believed that non-steroidal anti-inflammatory drugs such as Ibuprofen decrease the risk of COVID-19 complications. Only 28.6% knew that azithromycin is not safe to use with hydroxychloroquine to treat COVID-19 infection (S1 Table).

### Preparedness to deal with COVID-19

The preparedness score of all study participants ranged from 0 to 100%, with a mean of 68.65% (SD = 17.456) and a median of 68.00% (IQR = 24). Nearly a third of the participants (30.9%) had good preparedness scores, and 28.8% of the participants fell in the poor category.

**Fig 1:**
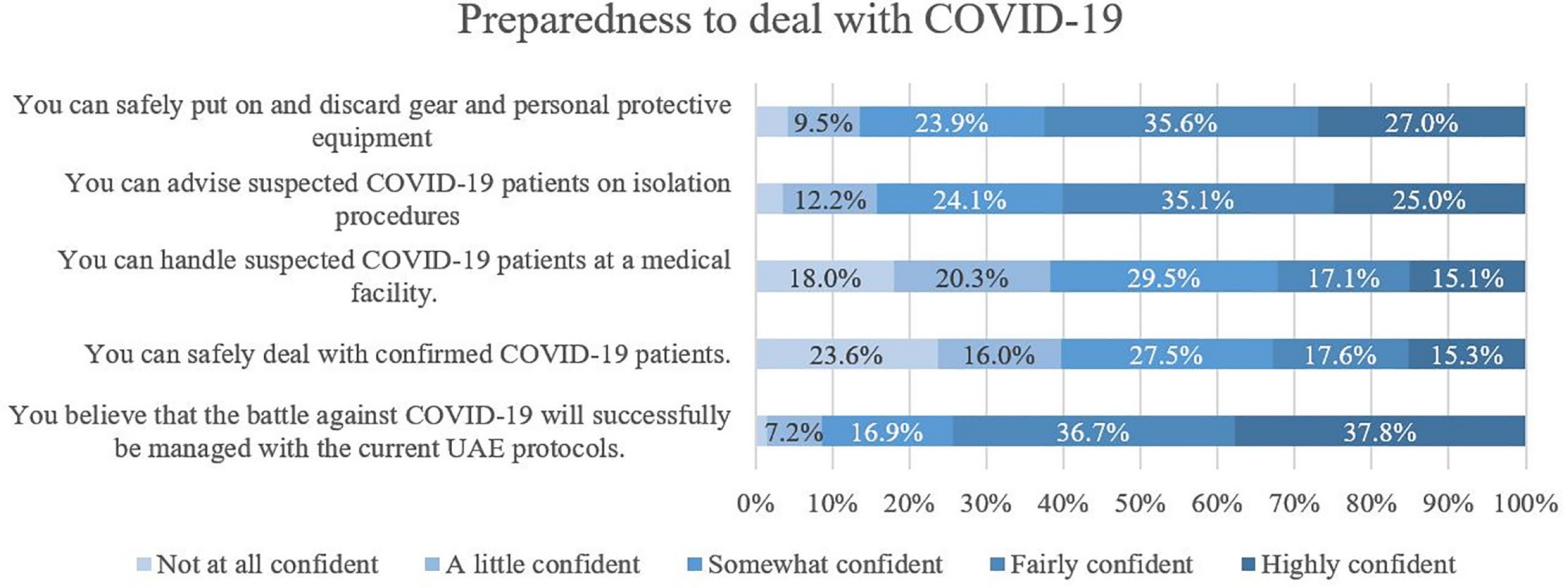
Sense of preparedness to serve in COVID-19 pandemic.

Participants who believed that college education had prepared them to deal with epidemics/pandemics had higher associated *(p<*0.001*)* preparedness scores as well as higher confidence in their COVID-19 knowledge *(p<*0.001*)*. Despite that, only 15.1% and 15.3% are highly confident about dealing with suspected and confirmed COVID-19 patients at a medical facility (Fig 1).

### Multivariate analysis of knowledge scores and preparedness scores

Table 2 outlines the significant associations observed between the dependent variables–knowledge score and preparedness score with the independent socio-demographic variables.

**Table 2:** Knowledge and preparedness among different socio-demographic sub-groups.

Variable	n	Knowledge Score		*p-value*	Preparedness Score		*p-value*
		Mean (Max = 25)	%		Mean (Max = 25)	%	
**Sex**				0.938 ^a^			**0.015**^a^
Female	298	14.78	59.12		16.81	67.24	
Male	146	14.75	59.00		17.88	71.52	
**Age Groups**				**<0.001**^b^			**<0.001**^b^
18–24	365	14.33	57.32		16.68	66.72	
25–35	36	16.47	65.88		19.81	79.24	
36–45	16	17.31	69.24		18.25	73.00	
>45	27	16.96	67.84		19.52	78.08	
**City of Residence**				**0.003**^b^			0.302^b^
Abu Dhabi	84	15.10	60.40		17.73	70.92	
Dubai	134	14.98	59.92		16.65	66.60	
Sharjah	192	14.82	59.28		17.32	69.28	
Northern Emirates	34	12.85	51.40		16.88	67.52	
**Nationality**				0.478^b^			0.136^c^
UAE National	89	14.42	57.68		18.04	72.16	
Arab Expatriate	278	14.83	59.32		16.94	67.76	
Non-Arab Expatriate	77	14.97	59.88		16.96	67.84	
**Professional Status**				**<0.001**^b^			**<0.001**^b^
Pre-clinical Phase Student (Years 1–3)	224	13.28	53.12		16.21	64.84	
Clinical Phase Student (Years 4–5)	80	15.04	60.16		15.73	62.92	
Recent Graduates/ Interns	65	16.94	67.76		19.22	76.88	
Resident Physician	15	17.67	70.68		21.60	86.40	
Physician/ Specialist/ Consultant	31	17.00	68.00		19.77	79.08	
Faculty Member	29	16.83	67.32		18.79	75.16	
**Years of clinical experience**				**<0.001**^b^			**<0.001**^b^
0 years	257	13.84	55.36		16.13	64.52	
1–5 years	146	15.82	63.28		18.36	73.44	
6–10 years	7	15.00	60.00		20.57	82.28	
> 10 years	34	17.24	68.96		19.12	76.48	
**Patient Contact**				**<0.001**^a^			**<0.001**^d^
Yes	131	16.57	66.28		19.39	77.56	
No	313	14.02	56.08		16.23	64.92	
**Patient Contact Frequency**				**0.045**^b^			0.728^c^
Everyday	56	16.93	67.72		20.04	80.16	
4–6 days per week	44	17.00	68.00		18.91	75.64	
1–3 days per week	23	15.09	60.36		19.09	76.36	
**Clinical Workshops Attended in Past Year**				**<0.001**^b^			**<0.001**^b^
None	116	13.16	52.64		16.12	64.48	
One	85	13.93	55.72		15.87	63.48	
Two	105	15.27	61.08		18.20	72.80	
Three	47	16.17	64.68		18.38	73.52	
Four	33	16.24	64.96		17.55	70.20	
Five	11	16.73	66.92		16.55	66.20	
> 5	47	16.28	65.12		18.40	73.60	

* *p*<0.05 is considered significant.

^a^Student’s T-test (parametric)

^b^One-sample ANOVA Test (parametric)

^c^Independent samples Kruskal-Wallis test (non-parametric)

^d^Mann-Whitney U test (non-parametric)

Stepwise multiple linear regression analysis models were designed for knowledge and preparedness scores, respectively to determine the predictive values of the variables.

The knowledge score model included the variables with significant associations *(p<*0.05*)* from Table 2: age-groups, city of residence, professional status, years of clinical experience, patient contact and workshops attended in the past year. The regression analysis yielded an adjusted R square of 0.384 and thus, the model was significant in predicting 38.4% of the total variability in the dependent knowledge score. (F = 38.033, *p* < 0.001).

As shown in Table 3, results of the model determined that occupation (*p*<0.001), being a resident of the Northern Emirates (*p*<0.001) and interest in attending at least one workshop (*p*<0.001) were significant and independent predictors of knowledge scores. The beta-coefficients highlight the degree of change in the dependent knowledge score for every unit change in the independent variable.

**Table 3:** Summary of stepwise multiple regression model for knowledge score.

Variable	Beta-coefficient	t-statistic	p-value	Semi-partial correlation
**Current Occupation**				
Pre-clinical Phase Student	-	-	-	-
Clinical Phase Student	0.194	4.593	**<0.001**	0.177
Recent graduate/ Interns	0.404	9.778	**<0.001**	0.377
Physician/ Specialist/ Consultant	0.282	6.989	**<0.001**	0.269
Faculty	0.277	6.872	**<0.001**	0.265
**City of Residence**				
Abu Dhabi	-	-	-	-
Dubai	-0.046	-1.149	0.251	-
Sharjah	0.019	0.468	0.640	-
Northern Emirates	-0.154	-3.970	**<0.001**	-0.153
**Workshops attended**				
None	-	-	-	-
At least one	0.186	4.508	**<0.001**	0.174

A similar model was designed for preparedness score, which included the significantly associated variables *(p<*0.05*)* from Table 2: sex, age, current profession, years of clinical experience, patient contact, workshops attended in the past year. The regression analysis yielded an adjusted R square of 0.172 and thus, the model was significant in predicting 17.2% of the total variability in preparedness score (F = 44.890, *p<0*.0005).

As shown in Table 4, results of the model determined that being in contact with patients (*p*<0.001) and being between the ages of 25–35 (*p*<0.030) were significant and independent predictors of preparedness scores.

**Table 4:** Summary of stepwise multiple regression model for preparedness score.

Variable	Beta-coefficient	t-statistic	p-value	Semi-partial correlation
**Patient Contact**				
No	-	-	-	-
Yes	0.387	8.543	**<0.001**	0.378
**Age**				
18–24 years	-	-	**-**	-
25–35 years	0.099	2.176	**0.030**	0.096
36–45 years	0.010	0.227	0.820	-
>45 years	Excluded from analysis due to collinearity			

### Attitudes and behaviours during the COVID-19 pandemic

All study participants’ attitude score ranged from 0 to 100%, with a mean of 74.89% (SD = 18.587) and a median of 83.33% (IQR = 17) as shown in Table 5.

**Table 5:** Attitudes during the COVID-19 pandemic.

Attitudes	Yes		No	
	n	%	n	%
When I meet my friends and colleagues, I will greet them with a handshake or a hug.	40	9.0	404*	91.0
If I come into contact with a person with symptoms, I will contact the health authorities.	200*	45.0	244	55.0
If I show symptoms similar to COVID-19, I will notify the health authorities.	365*	82.2	79	17.8
I will wash my hands with soap and water for 20 seconds after contact with every patient or contact with their surroundings.	397*	89.4	47	10.6
I will only wear a face mask when I am in the company of somebody with flu-like symptoms.	81	18.2	363*	81.8
It is safe for me to go to any public place if I wear a mask.	178	40.1	266*	59.9

* indicates the favourable attitude.

Attitude scores were found to be significantly different across different sexes *(p<*0.001), age groups *(p =* 0.036*)*, and cities of residence *(p<*0.001*)*. In contrast to knowledge score associations, Northern Emirates had the highest attitude scores as opposed to other emirates, and the age group 18–24 years, had more favourable attitude scores. Also, females showed a higher attitude score in comparison to males. An alarming finding was that 18.2% of participants mentioned that they would only wear a face mask when in the company of someone with flu-like symptoms.

### Sources of COVID-19 knowledge

**Fig 2:**
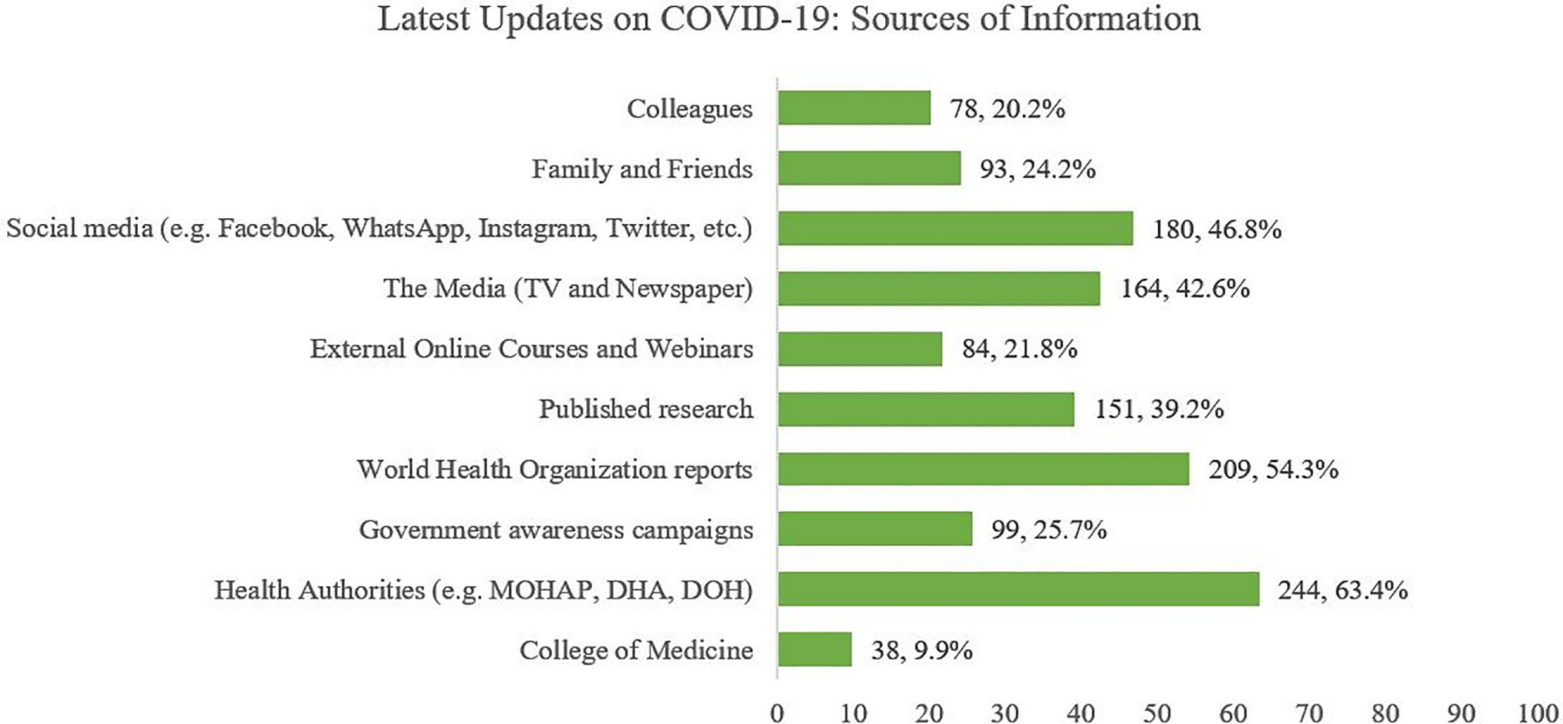
Sources of information on COVID-19.

The participants were asked whether they follow the latest updates on COVID-19, and 86.7% answered "Yes". The sources used by the participants are demonstrated in Fig 2. The most common sources used were Health Authorities (63.4%), followed by WHO reports (54.3%), and Social Media (46.8%).

The mean number of sources used by each participant was 3.48 (SD = 2.09). A significant association was observed between knowledge score, and the number of sources used to obtain information on COVID-19 (*p<*0.001*)*.

**Fig 3:**
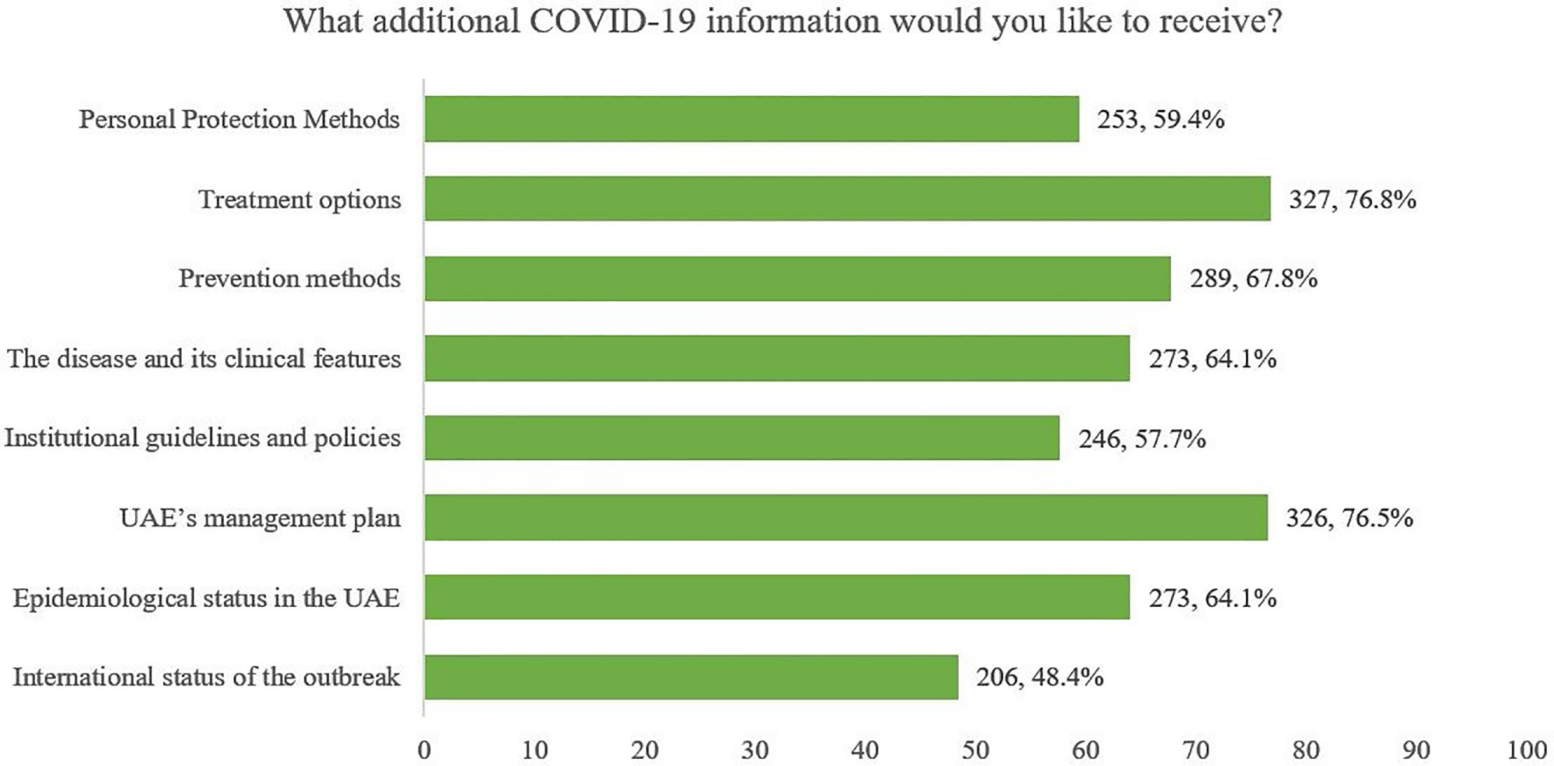
Additional COVID-19 information participants would like to receive.

Additionally, the participants were asked if they are satisfied with their current knowledge on COVID-19 and whether they would want to receive further information. 81.31% were not satisfied with their current information and required further information on the disease, as described in Fig 3.

### Perceptions regarding the roles of colleges of medicine and health authorities

Participants’ perception on this topic was measured using nine questions, as outlined in S2 and S3 Tables. Most participants agreed that the Colleges of Medicine (63.5%) and Health Authorities (83.1%) have a role in preparing them to deal with future epidemics and pandemics. Furthermore, 84% also agreed that both these should join forces and produce one educational module for the management of the COVID-19 pandemic, and that they should contribute to knowledge on COVID-19 through sending awareness emails, online workshops, and lectures, and providing information resources.

### Barriers and incentives affecting willingness to work in current and future pandemics

**Fig 4:**
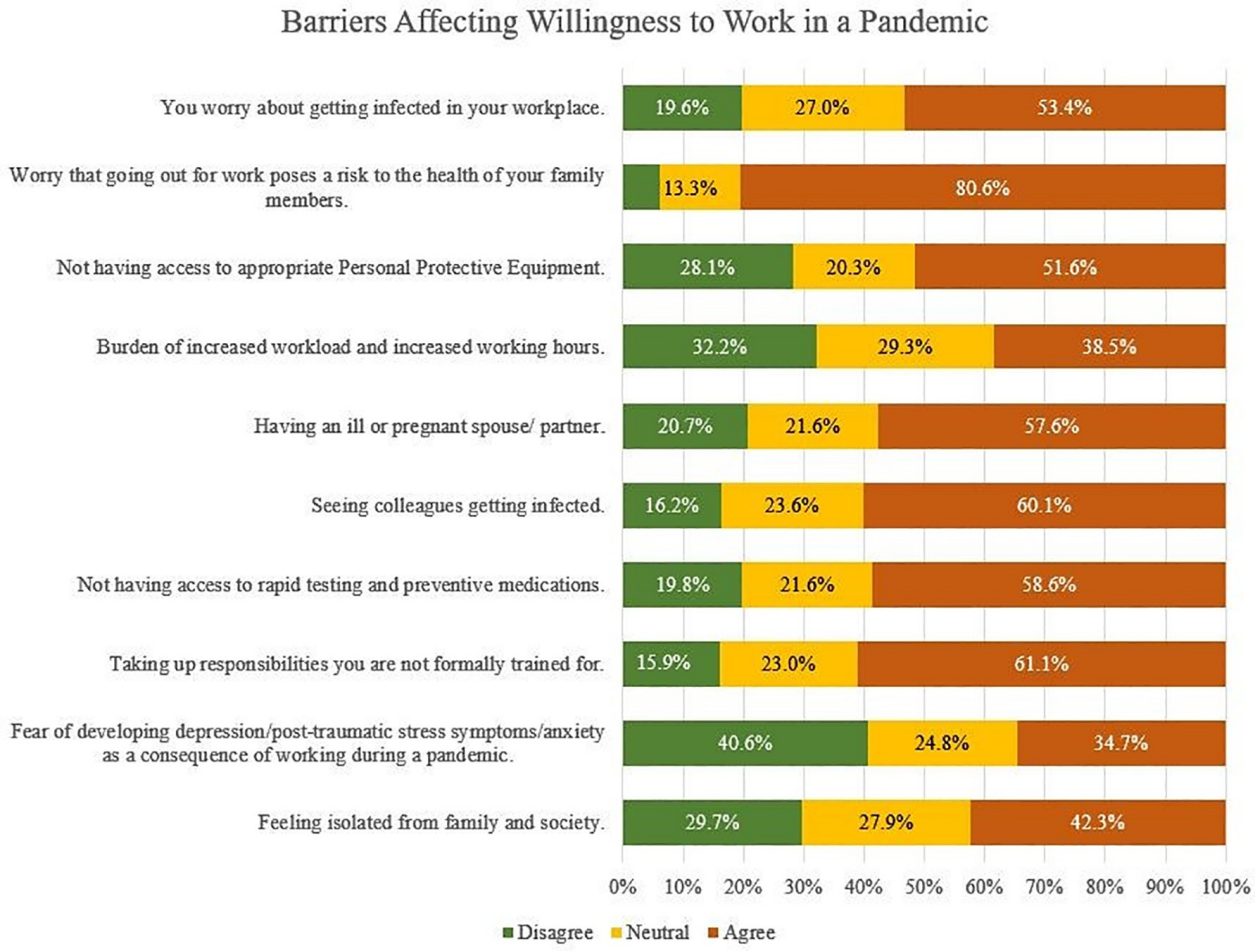
Barriers affecting willingness to work in a pandemic.

In the final section of the questionnaire, the participants were asked to state their level of agreement to 18 statements describing barriers and incentives which could affect their willingness to work during a pandemic (Fig 4). Barriers and incentives were initially asked in the form of the 5-item Likert scale and then collapsed for the sake of results representation.

Amongst all the barriers, participants were mostly anxious about the increased risk of transmission to family members due to their profession (80.6%), and not being able to fully support an ill or pregnant partner (57.6%). Seeing other colleagues getting infected while at work was also a major source of concern (60.1%). Parallely, 77% stated that feeling protected by their employers as well as access to frequent screening (80.9%) were the most important incentives to report to work during a pandemic. Results also indicated that most participants agreed that incentives such as receiving bonus pay (60.4%), feeling protected by health authorities (77.0%), receiving family support (77.0%), and frequent screening for infection at the workplace (80.9%) would increase their willingness to work during a pandemic. Further details attached in S4 Table.

## Discussion

COVID-19 has emerged as a strong catalyst for change all around the world, significantly challenging all sectors. Now more than ever, the world is turning to the leaders in the field of medicine for their support and guidance. This puts medical students, physicians, and faculty at the forefront against this pandemic. Unfortunately, they are also amongst the ones who are at higher risk of acquiring the disease compared to the rest of the population and therefore, it is vital that they have adequate knowledge about all aspects of the disease and feel prepared to take on their responsibilities.

At the time of designing and conducting this study, the United Arab Emirates experienced its first major peak of COVID-19 cases between May 2020 and July 2020. The results and perspectives reported are representative of the situation then.

The knowledge and attitudes of healthcare workers across the world, including the UAE, was explored at the onset of the crisis in March 2020 by Bhagavathula *et al*. [14]. However, to the best of our awareness, this is the first study that explores the variables amongst medical students and physicians exclusively based in the United Arab Emirates. Furthermore, it is also the first study to explore the barriers affecting willingness to work during the current pandemic and recommendations to prepare for future pandemics in the country.

### Knowledge about COVID-19 as a disease is insufficient

In this study, the mean knowledge score of the participants was found to be 59.08%. However, only 7.9% had a knowledge score equal to 80% or above. Owing to increased awareness with time, these findings are an improvement from the poor knowledge of symptoms and transmission in a similar population in the UAE, reported by Bhagavathula *et al*. earlier in the year [14].

However, the knowledge score found in this study is lower than those of similar studies conducted amongst health care workers around the world, with scores of 69% from Uganda [15], 88.4% from Vietnam [16], 80.4% from Egypt [17] and 93.2% from Pakistan [18]. These comparisons must not be taken as absolute without consideration of the differences in questionnaire tools. In the absence of a validated tool to assess COVID-19 knowledge, most of the above-mentioned studies used self-designed questions focusing on symptoms, transmission, and precautions. Since this questionnaire was particularly directed to medical students and professionals, it is expected that their knowledge would be more esoteric than that of the general population. Hence, a more comprehensive tool was developed that also focuses on awareness regarding laboratory findings, complications of disease and management guidelines which is where the majority answered incorrectly, resulting in a poorer total knowledge score.

### Sources of information about COVID-19 are varied and diverse

The findings of this study coincide with a study conducted in Uganda [19]: WHO reports (88%), governmental health authorities (79%) and social media (74%), which also indicate a significant use of both the health authorities and social media as primary sources of information amongst participants. However, the extent to which participants used social media as the main platform differed in this study (46.8%) than an 83.4% use seen in a study conducted in Jordan amongst medical students [19]. Furthermore, a cross-sectional study conducted in Pakistan [18] noted that 87.68% of healthcare practitioners used social media as the main source of information. Knowing that participants are using social media more than the College of Medicine (9.9%) and published research (39.2%) is a common finding which is consistent in the United Arab Emirates as well. It may be quite challenging for young medical students to track scientific information from large databases and thus, it would be beneficial if medical schools could educate and advise them on how to search for high-quality data from such repositories.

### Colleges of medicine and health authorities should play an important role during a pandemic

When preparing medical students and personnel for the frontline during a pandemic/epidemic, it is important that they receive information from reliable sources such as the Colleges of Medicine and Health Authorities, as they play a major role in building the knowledge of medical students from its preliminary stages. These institutions play an integral role in forming the basis of medical studies and information distribution of which is relevant to time and country of residence.

This is also further compounded by the fact that COVID-19 knowledge has a significant relationship with age-group and professional status respectively, implying that seniority in age and experience translated to better knowledge outcomes unlike other studies where knowledge about COVID-19 was not associated with age [16]. With a rise in medical personnel demand during the pandemic, it has been inevitable to observe the participation of far less experienced healthcare providers such as medical students and interns.

A large proportion of the population agreed that colleges have a role in equipping students to deal with future epidemics/pandemics but only 9.9% of the population identified the Colleges of Medicine as a current source of information for COVID-19 updates. This highlights a significant gap between demand and supply and may be an indicator to develop engaging courses with targeted information catering to the demographic. Mohammed Bin Rashid University of Medicine and Health Sciences, one of UAE’s many medical universities, took the first step in this regard and launched several community-based awareness courses, with the endorsement of the national health ministries. Similarly, increased efforts are needed to improve preparedness to deal with COVID-19.

### Population perceives that previous medical education is not adequate to deal with epidemics or pandemics

The study also investigated participants’ perception of prior epidemics. Although, the majority agreed that their respective Colleges of Medicine had been their primary sources of information about previous epidemics/pandemics, only 27.9% of the total participants agreed that their college education has provided adequate knowledge to deal with epidemics or pandemics. It is worth noting that higher levels of COVID-19 knowledge scores were observed in participants who have reported to receive little previous education about pandemics/epidemics and vice versa. This may shed light on the self-directed learning that most people have had to undertake in the absence of adequate guidance from their respective educational institutions.

### Multiple barriers affect willingness to work during a pandemic

There is an increased strain on the health workforce during periods of epidemics and pandemics, and the challenge becomes two-fold; ensuring that the community is safe and cared for and that the workforce is maintained and their ability to care for patients is augmented. In such a situation, it is important to identify the potential sources of anxiety that may hamper current and future doctors’ confidence and potential.

The findings of this study are in line with similar investigations carried out during the time of H1N1 pandemic in 2009. In a study conducted in the United States, 80% of essential workers reported they would be able to report to duty but only about 65% would be willing to do so [9]. Major factors associated with willingness depended on personal adherence to respiratory protection as well as organisational preparedness [9]. Garret *et al*., in their study on ’Mitigating Absenteeism in Hospital Workers during a Pandemic’ identified that interventions that included the employee’s immediate family had a significantly greater impact than those that included the employee alone, such as additional monetary compensation or leave [20].

The United Arab Emirates has taken several steps to make their frontline workers feel valued and cared for during the pandemic by offering incentives in the form of salary bonuses [21]. Children of frontline workers have also been offered scholarships [22], amongst other governmental measures. While these are valuable and appreciated, this study’s findings have identified that institutions need to develop a comprehensive plan that strongly stands behind their healthcare workers during crises.

### Limitations of the study

Although this paper is based on a comprehensive questionnaire and significant results, it is important to understand its limitations. First and foremost, it used convenience sampling methods which may affect the generalizability of the results. It is also an internet-based questionnaire and may be affected by a selection bias. People may have volunteered to participate because of increased knowledge or perception and may over-represent some groups of the population. Furthermore, the distribution was carried out mainly on social networking platforms, which has an increased presence of younger students than more senior professionals, leading to disproportionate sample distribution, combined with a relatively small sample size of only 444 participants.

Additionally, this study was conducted from May to July 2020, which was during the final weeks of lockdown in the UAE; during this time period, colleges and health authorities had limited information on the pandemic and limited time for data distribution; this may have affected the preparedness and knowledge scores of the studied population. The findings may thus, under-represent the magnitude of awareness of COVID-19 and behaviours associated with it in the chosen population.

These results may also be representative of other countries in the Middle East with a similar socioeconomic status. However, differences in COVID-19 cases and healthcare settings around the world may affect the global generalizability of the results.

Further studies discussing healthcare attitudes and preparedness in context of the COVID-19 pandemic may be warranted with a randomized study design and a larger population size. Interesting avenues for future studies could cover more recent updates in the knowledge and attitudes of physicians as well as strategies to design curricula catering to pandemic preparedness in medical schools across UAE.

## Conclusion

Medical students and physicians undeniably play a critical role in dealing with current and future pandemics. This study sheds light on the adequacy of their knowledge, preparedness, and behaviours in dealing with the COVID-19 pandemic. Most participants showed inadequate knowledge, which establishes a need to intensify knowledge resources. Since participants in older age groups and higher clinical experience attained higher knowledge scores, increased efforts need to be focused particularly towards pre-clinical medical students. However, most participants showed adequate preparedness and attitudes scores. In this time of crisis, studies such as these, along with the extensive efforts of the United Arab Emirates government, serve as an essential contribution to fill the gaps on and eventually defeat the challenges presented by COVID-19.

### Participant Information Sheet (PIS)

The PIS was presented before starting the questionnaire. Agreement to fill the questionnaire indicated the agreement of the participants to join the study; this was clearly mentioned in the PIS.

## Supporting information

S1 Table Knowledge questions about COVID-19. (DOCX)

S2 Table Role of colleges of medicine in pandemic training. (DOCX)

S3 Table Role of health authorities in pandemic training. (DOCX)

S4 Table Incentives which increase willingness to work in current or future pandemics. (DOCX)
