# Supplementary material for: Readiness of physicians and medical students to cope with the COVID-19 pandemic in the UAE
Source: PLoS One. 2021 May 6;16(5):e0251270. doi: 10.1371/journal.pone.0251270 (PMC8101710; doi:10.1371/journal.pone.0251270)
Supplement: S1 Table — (DOCX) [file pone.0251270.s001.docx]

**S1 Table: Knowledge Questions about COVID-19**

| Questions | True | | False | | I do not know | |
| --- | --- | --- | --- | --- | --- | --- |
|  | Number | % | Number | % | Number | % |
| What are the common symptoms observed in COVID-19? (Choose all that apply) | | | | | | |
| Fever | 436* | 98.2 | 8 | 1.8 | - | - |
| Cough | 428* | 96.4 | 16 | 3.6 | - | - |
| Anorexia | 64* | 14.4 | 380 | 85.6 | - | - |
| Seizures | 7 | 1.6 | 437* | 98.4 | - | - |
| Constipation | 23 | 5.2 | 421* | 94.8 | - | - |
| Shortness of Breath | 421* | 94.8 | 23 | 5.2 | - | - |
| Myalgia | 172* | 38.7 | 272 | 61.3 | - | - |
| Bloody Diarrhoea | 17 | 3.8 | 427* | 96.2 | - | - |
| Sputum production | 67* | 15.1 | 377 | 84.9 | - | - |
| Skin Infection | 14 | 3.2 | 430* | 96.8 | - | - |
| Fatigue | 341* | 76.8 | 103 | 23.2 | - | - |
| I do not know |  |  |  |  | 3 | 0.7 |
| What are the routes of human-to-human transmission of COVID-19? (Choose all that apply) | | | | | | |
| Exposure of nose / mouth to infected respiratory droplets | 430* | 96.8 | 14 | 3.2 | - | - |
| Aerosol generating procedures (Bronchoscopy/Endotracheal intubation/ CPR) | 250* | 56.3 | 194 | 43.7 | - | - |
| Sexual transmission | 24 | 5.4 | 420* | 94.6 | - | - |
| Feco-oral transmission | 44 | 9.9 | 400* | 90.1 | - | - |
| From pregnant mother to child (transplacental) | 59 | 13.3 | 385* | 86.7 | - | - |
| Exposure of eyes/ conjunctiva to infected respiratory droplets | 309* | 69.6 | 135 | 30.4 | - | - |
| The transmission is not well understood | 92* | 20.7 | 352 | 79.3 | - | - |
| I do not know |  |  |  |  | 6 | 1.4 |
| Answer the following statements with True, False, or I do not know. | | | | | | |
| Incubation period of SARS-COV-2 | | | | | |  |
| The incubation period of COVID-19 (time between infection and appearance of clinical symptoms) can last from 2-14 days. | 426* | 95.9 | 10 | 2.3 | 8 | 1.8 |
| Signs and Symptoms of COVID-19 | | | | | | |
| Similar symptoms are experienced in all infected patients, regardless of medical condition. | 60 | 13.5 | 359* | 80.9 | 25 | 5.6 |
| All patients presenting to the hospital with flu like symptoms have to be tested for COVID-19. | 328 | 73.9 | 81* | 18.2 | 35 | 7.9 |
| Not all persons with COVID-19 will develop to severe cases. Only those who are elderly, have chronic illnesses, and are obese are more likely to be severe cases. | 378* | 85.1 | 50 | 11.3 | 16 | 3.6 |
| Laboratory Results | | | | | | |
| A positive antibody test tells you that the patient has a current infection. | 154 | 34.7 | 245* | 55.2 | 45 | 10.1 |
| A negative COVID-19 lab test (RT-PCR) indicates that the patient is free of the insulting pathogen. | 264 | 59.5 | 142* | 32.0 | 38 | 8.6 |
| Lymphopenia is a characteristic lab finding of COVID-19. | 160* | 36.0 | 77 | 17.3 | 207 | 46.6 |
| Complications of COVID-19 | | | | | | |
| Acute Respiratory Distress Syndrome (ARDS) and acute tubular necrosis are common complications of COVID-19. | 315* | 70.9 | 34 | 7.7 | 95 | 21.4 |
| Prevention Methods | | | | | | |
| Handwashing with soap and water for 20 seconds is enough to clean the hands and protect from spreading the infection. | 318* | 71.6 | 100 | 22.5 | 26 | 5.9 |
| You need to keep a distance of at least 3 meters (10 feet) when counselling patients during a pandemic. | 264 | 59.5 | 126* | 28.4 | 54 | 12.2 |
| Respirator (N95 mask) and gloves are sufficient when dealing with suspected or confirmed COVID-19 patients. | 179 | 40.3 | 229* | 51.6 | 36 | 8.1 |
| Management | | | | | | |
| Non-steroidal anti-inflammatory drugs such as Ibuprofen decrease the risk of COVID-19 complications. | 64 | 14.4 | 251* | 56.5 | 129 | 29.1 |
| Azithromycin is safe to use with Hydroxychloroquine to treat the COVID-19 infection. | 133 | 30.0 | 127* | 28.6 | 184 | 41.4 |
| There is no current approved treatment for COVID-19 management is symptomatic in most cases. | 391* | 88.1 | 30 | 6.8 | 23 | 5.2 |
| * indicates correct answer | | | | | |  |
