# Supplementary material for: Readiness of physicians and medical students to cope with the COVID-19 pandemic in the UAE
Source: PLoS One. 2021 May 6;16(5):e0251270. doi: 10.1371/journal.pone.0251270 (PMC8101710; doi:10.1371/journal.pone.0251270)
Supplement: S2 Table — (DOCX) [file pone.0251270.s002.docx]

**S2 Table: Role of Colleges of Medicine in Pandemic Training**

| Statement | Strongly disagree | | Somewhat disagree | | Neutral | | Somewhat agree | | Strongly agree | |
| --- | --- | --- | --- | --- | --- | --- | --- | --- | --- | --- |
|  | n | % | n | % | n | % | n | % | n | % |
| The College has a role in preparing you to deal with future epidemics/ pandemics | 16 | 3.6 | 46 | 10.4 | 100 | 22.5 | 105 | 23.6 | **177** | **39.9** |
| The College should incorporate epidemics/ pandemics management course into their syllabus. | 5 | 1.1 | 16 | 3.6 | 72 | 16.2 | 94 | 21.2 | **257** | **57.9** |
| The College should provide online lectures and webinars to students and alumni | 4 | 0.9 | 24 | 5.4 | 73 | 16.4 | 97 | 21.8 | **246** | **55.4** |
| The College should provide online educational workshops on the COVID-19 pandemic | 4 | 0.9 | 18 | 4.1 | 78 | 17.6 | 101 | 22.7 | **243** | **54.7** |
| The College should provide online information resources (e.g. summary of research studies) on the COVID-19 pandemic. | 2 | 0.5 | 7 | 1.6 | 68 | 15.3 | 91 | 20.5 | **276** | **62.2** |
