# Supplementary material for: Readiness of physicians and medical students to cope with the COVID-19 pandemic in the UAE
Source: PLoS One. 2021 May 6;16(5):e0251270. doi: 10.1371/journal.pone.0251270 (PMC8101710; doi:10.1371/journal.pone.0251270)
Supplement: S3 Table — (DOCX) [file pone.0251270.s003.docx]

**S3 Table: Role of Health Authorities in Pandemic Training**

| Statement | Strongly disagree | | Somewhat disagree | | Neutral | | Somewhat agree | | Strongly agree | |
| --- | --- | --- | --- | --- | --- | --- | --- | --- | --- | --- |
|  | n | % | n | % | n | % | n | % | n | % |
| The Health Authorities have a role in preparing you to deal with epidemics/ pandemics. | 6 | 1.4 | 8 | 1.8 | 61 | 13.7 | 90 | 20.3 | **279** | **62.8** |
| The Health Authorities should send awareness emails on the current pandemic | 2 | 0.5 | 21 | 4.7 | 50 | 11.3 | 108 | 24.3 | **263** | **59.2** |
| The Health Authorities should provide online educational workshops on the COVID-19 pandemic. | 3 | 0.7 | 9 | 2.0 | 51 | 11.5 | 110 | 24.8 | **271** | **61.0** |
| The Colleges of Medicine and Health Authorities should join forces and produce one educational module for the management of the COVID-19 pandemic. | 1 | 0.2 | 8 | 1.8 | 62 | 14.0 | 84 | 18.9 | **289** | **65.1** |
