# Supplementary material for: Readiness of physicians and medical students to cope with the COVID-19 pandemic in the UAE
Source: PLoS One. 2021 May 6;16(5):e0251270. doi: 10.1371/journal.pone.0251270 (PMC8101710; doi:10.1371/journal.pone.0251270)
Supplement: S4 Table — (DOCX) [file pone.0251270.s004.docx]

**S4 Table: Incentives which Increase Willingness to Work in Current or Future Pandemics**

| Statement | Strongly disagree | | Somewhat disagree | | Neutral | | Somewhat agree | | Strongly agree | |
| --- | --- | --- | --- | --- | --- | --- | --- | --- | --- | --- |
|  | n | % | n | % | n | % | n | % | n | % |
| Receiving bonus pay as compensation. | 32 | 7.2 | 40 | 9.0 | 104 | 23.4 | 102 | 23.0 | **166** | **37.4** |
| Feeling protected by health authorities and employers. | 5 | 1.1 | 12 | 2.7 | 85 | 19.1 | 95 | 21.4 | **247** | **55.6** |
| Receiving adequate childcare and family support from authorities. | 6 | 1.4 | 11 | 2.5 | 85 | 19.1 | 105 | 23.6 | **237** | **53.4** |
| Access to psychological support during and after the pandemic. | 14 | 3.2 | 35 | 7.9 | 96 | 21.6 | 102 | 23.0 | **197** | **44.4** |
| Frequent screening for infection at the workplace. | 8 | 1.8 | 11 | 2.5 | 66 | 14.9 | 86 | 19.4 | **273** | **61.5** |
| Working for two weeks followed by a period of self-isolation for two weeks. | 23 | 5.2 | 33 | 7.4 | 135 | 30.4 | 82 | 18.5 | **171** | **38.5** |
| Access to updated information and courses. | 3 | 0.7 | 20 | 4.5 | 77 | 17.3 | 101 | 22.7 | **243** | **54.7** |
| Community privileges such as discounts, reduced transport fees etc. | 26 | 5.9 | 23 | 5.2 | 87 | 19.6 | 104 | 23.4 | **204** | **45.9** |
